# The role of routine transpedicular biopsies during kyphoplasty or vertebroplasty for vertebral compression fractures in the detection of malignant diseases: a systematic review

**DOI:** 10.1007/s00402-022-04392-7

**Published:** 2022-03-01

**Authors:** Georg Osterhoff, Max J. Scheyerer, Ulrich J. A. Spiegl, Klaus J. Schnake

**Affiliations:** 1 Department of Orthopaedics, Trauma and Plastic Surgery, University Hospital Leipzig, 04103 Leipzig, Germany; 2 Department of Orthopedics and Trauma Surgery, University Hospital of Cologne, Cologne, Germany; 3 Center for Spinal and Scoliosis Surgery, Malteser Waldkrankenhaus St. Marien, Erlangen, Germany; 4 Department of Orthopedics and Traumatology, Paracelsus Private Medical University Nuremberg, Nuremberg, Germany

**Keywords:** Spine, Tumors, Infections, Pathological fracture, Biopsy, Vertebroplasty, Kyphoplasty

## Abstract

**Introduction:**

Procedures like kyphoplasty or vertebroplasty have become an established treatment option for vertebral compression fractures (VCF). The transpedicular approach used during these procedures allows to take biopsies from the affected vertebral body. The aim of this study was to systematically summarize the existing knowledge on the value of routine transpedicular biopsies during kyphoplasty or vertebroplasty for vertebral compression fractures.

**Methods:**

A systematic review of the literature using PubMed/Medline databases with the goal of finding all articles describing the value trans-pedicular biopsies for detecting primary bone tumors, metastases, bone diseases, or spondylitis in patients with vertebral compression fractures was performed. Search terms were (*biopsy/ OR biops*.ti,ab.) AND (vertebral compression fracture*.ti,ab.).

**Results:**

Sixteen articles met the inclusion criteria, among these were six prospective and ten retrospective case series. Publication dates ranged from 2005 to 2020. A total of 3083 patients with 3667 transpedicular biopsies performed were included. Most biopsies confirmed osteoporosis as the dominant underlying pathology of VCFs. Transpedicular biopsies revealed an unexpected malignant diagnosis in 0.4–6% of the cases.

**Conclusion:**

Routine transpedicular biopsies during kyphoplasty or vertebroplasty detect unexpected malignant lesions in 0.4–6% of the patients, even though the definition of “unexpected” varies among the analyzed studies. The evidence to support a routine biopsy is inconsistent. Nevertheless, routine biopsies can be considered, especially when sufficient preoperatvie imaging is not available or radiological findings are unclear.

## Introduction

Pathologic fractures are defined as fractures with no or inadequate previous trauma. They can be caused by any disease that weakens the architecture of bones, osteoporosis and metastatic disease being the most frequent one [1, 2]. The spine, in particular, is a common localization of bone metastases and multiple myeloma lesions that together with osteoporosis all can result in pathologic vertebral compression fractures (VCF) [3, 4].

While in most cases, metastatic and osteoporotic fractures can be differentiated by the patient’s history and the imaging findings, missing an underlying malignant disease can be fraught with consequences for the patient.

Vertebral augmentation procedures like kyphoplasty or vertebroplasty have become an established treatment option for vertebral compression fractures [5]. The transpedicular approach used during these procedures easily allows a large caliber needle biopsy of the fractured vertebral body in all patients. However, obtaining a biopsy in every patient takes time and is associated with increased costs for the biopsy needle and for histopathological assessment.

Several studies investigated the prevalence of previously unrecognized primary tumors or metastatic disease in this patient group. The aim of this study was to systematically summarize the existing knowledge on the value of transpedicular biopsies in the detection of malignant diseases when performed during kyphoplasty or vertebroplasty for vertebral compression fractures. It was the authors’ hypothesis that the prevalence of unexpected malignancies is higher than presumed by most surgeons.

## Materials and methods

### Study design

We conducted a systematic review of the literature according to the PRISMA (Preferred Reporting Items for Systematic Reviews and Meta-analyses) checklist and algorithm [6], with the goal of finding all articles describing the value trans-pedicular biopsies for detecting primary bone tumors, metastases, bone diseases or spondylitis in patients with vertebral compression fractures.

### Search strategy

**Fig. 1:**
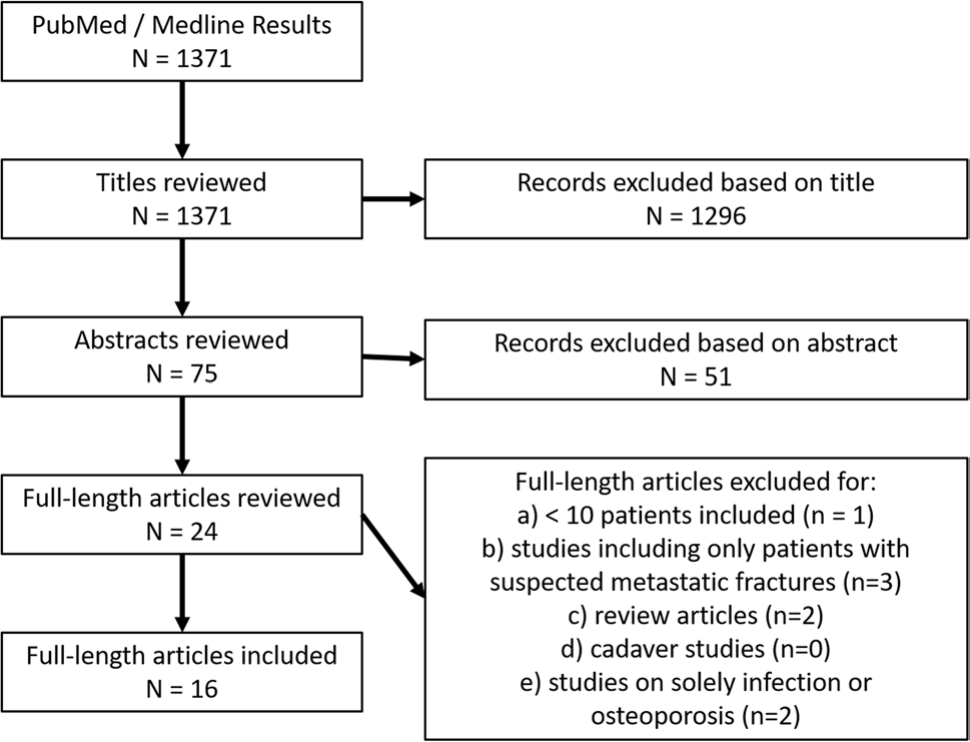
Screening flow chart

Investigations between 2000 and 2020 were included. Prospective and retrospective observational investigations were considered for analysis. The authors performed a systematic an initial search of PubMed/Medline databases for eligible investigations. The search terms were (*biopsy/ OR biops*.ti,ab.) AND (vertebral compression fracture*.ti,ab.)*. *The authors limited the research to observational studies, while systematic reviews, meta-analyses, case series and case reports were excluded. Titles and abstracts were reviewed. Duplicates were removed and full texts were checked for suitability. The remaining abstracts and full-length articles were then screened by all authors and disagreement was resolved by consensus. The final decision was made based on the analysis of the full text. In doubt, articles were included into the next stage. A flowchart of the filtering stages (titles, abstracts, full-length texts) is shown in Fig. 1.

Studies were selected according the following inclusion criteria: (a) case series, cohort studies, or clinical trials regarding biopsies in patients with vertebral compression fractures, (b) trans-pedicular biopsy during vertebroplasty or kyphoplasty or similar trans-pedicular therapeutic intervention, (c) biopsy within 3 months of the fracture, (d) histological work-up documented, (e) articles published in English or German language. Exclusion criteria were: (a) case reports or case series with less than 10 patients, (b) studies that on purpose included patients with suspected metastatic fractures based on the patients’ cancer history, (c) review articles, (d) cadaver studies, (e) studies that did not report on malignant lesions at all (i.e., studies on solely infection or osteoporosis).

### Synthesis of results

We extracted data concerning study characteristics including authors’ names, title, year of publication, journal of publication, number of patients, time of follow-up and type of study. For the description of the study population, number of patients and age were collected. Outcome parameters were analyzed according to the inclusion criteria. For all included studies, we used the Oxford Centre for Evidence-Based Medicine 2011 for defining the level of evidence [7].

## Results

Sixteen articles met the inclusion criteria (Fig. 1), among these were six prospective (Table 1) and 10 retrospective case series (Table 2). Publication dates ranged from 2005 to 2020 and included 42 different authors. A total of 3083 patients with 3667 transpedicular biopsies performed were included.

**Table 1:** Prospective studies

Year	Authors	Journal	Level of evidence	Patients (*n*)	Biopsies (*n*)	Mean age (y)	Imaging	Osteology (%)	Malignancy (%)	Osteomyelitis (%)
2005	Togawa	SPINE	I	142	178	72, 40 to 90	Not reported	100% bone remodeling and/or fracture-healing, 21% increased osteoid, suggesting either increased bone-remodeling or osteomalacia	2%	–
2006	Shindle	J Bone Joint Surg Am	I	238	523	–	MRI	100% bone-remodeling and/or fracture-healing	2%	
2010	Pneumaticos	Eur Spine	I	75	154	69, 32 to 87	bone scan + MRI	100% variable amounts of unmineralised bone, signs of bone-remodeling and/or fracture-healing	15% 1% unexpected	–
2013	Zhang	Oncol Lett	I	546	692	75, 56 to 93	MRI	73% osteoporosis	5%, 0.4% unexpected	–
2014	Mukherjee	J Neurosurg Spine	I	147	184	–	MRI	–	6%, 5% unexpected	–
2014	Li	PLOS ONE	I	97	151	69 ± 10	MRI	1% M. Paget	3% (all unexpected)	–

**Table 2:** Retrospective studies

Year	Authors	Journal	Level of evidence	Patients (*n*)	Biopsies (*n*)	Mean age (y)	Imaging	Osteology (%)	Malignancy (%)	Osteomyelitis (%)
2007	Libicher	Eur Radiol	IV	180	180	63 ± 12	CT	75% osteoporosis 7% osteonecoris	11%	–
2008	Schoenfeld	Injury	IV	50	80	76	CT		8%, 6% unexpected	
2009	Allen	SPINE	IV	66	94	73, 22 to 99	MRI	“Majority” showed different stages of healing in osteoporotic fractures	5%	6.4%
2009	Muijs	SPINE	IV	78	71	73, 48 to 93	MRI	54% “reactive changes due to bone regeneration, growth, and remodeling”	4% (all unexpected)	–
2016	Hansen	SPINE	IV	144	137	73, 56 to 92	MRI	–	6% (all unexpected)	–
2016	Pagdal	Asian Spine J	IV	84	79	66	MRI	87% osteoporosis	10%, 1% unexpected	–
2018	Nowak	Eur Spine J	IV	97	97	68, 18 to 89	Bone scan + MRI		10%, 3% unexpected	0%
2019	Zhihong	Int J Spine Surg	IV	410	410	74, 26 to 96	MRI	–	11% 1% unexpected	–
2019	Uzunoglu	World Neurosurg	IV	533	505	62	MRI	100% various stages of bone healing, < 1% M. Paget	5%, 1% unexpected	1%
2020	Hershkovich	SPINE	IV	116	122	77, 52 to 92	Bone scan / MRI	–	20%, 1% unexpected	–

One of the largest series dealing with the significance of a biopsy during kypho- or vertebroplasty was done by Zhang et al. [8]. The authors analyzed 692 biopsies from 546 patients. The samples were collected during vertebroplasties or kyphoplasties. The histological results of 398 patients were in good agreement with the diagnosis of osteoporotic VCFs. Among 44 patients with a history of malignancy, the pathology could be verified in 25 patients. In two cases a previously undiagnosed malignancy (one multiple myeloma, one metastatic carcinoma) came to light. In a prospective series of 692 biopsies by Zhang et al., only 0.4% unsuspected malignancies were found [8]. Other investigations reported significantly higher incidence rates of unsuspected malignancy in biopsies during vertebroplasty and came to similar conclusions [9]. Hansen et al., Nowak et al., Li et al., and Uzunoglu et al. observed an incidence of 4.9%, 3.1%, 2.9%, and 1.1% respectively [10–13]. Based on their results, Li et al. further analyzed the economic burden due to routine biopsy. They concluded that a biopsy in every vertebroplasty or kyphoplasty is cost-effective in finding new malignancies compared to routine cancer screening campaigns [12].

In a retrospective case series, Allen et al. examined 94 vertebral biopsies from 66 patients during kyphoplasty for VCFs. Biopsies predominantly showed benign-appearing bone with histologic features consistent with osteoporotic VCFs in multiple stages of healing [14]. However, four biopsies (4.2%) helped to confirm a suspected malignancy or to rule out the recurrence of malignant disease. The authors advocate for a routine bone biopsy during kyphoplasty in all patients undergoing first-time vertebral augmentation. Beside secondary malignant processes, Muijs et al. reported about the detection of previously undiagnosed primary malignancies in 3 patients (3.8% of all biopsies), whereby two multiple myeloma stage IIa and one chondrosarcoma grade I were found [15]. In one retrospective observational study conducted by Pagdal et al. involving 84 patients [16], primary spinal malignancies were seen in some cases. Malignancy could be detected in 8 patients. Among these eight patients were two patients with metastases and six patients with a primary spinal malignancy (1 unsuspected plasmacytoma, 5 already suspected from preoperative imaging). In their investigation, the frequency of unsuspected malignancy was 1.2% [16].

But also in cancer patients suspected of being in remission, a new cancer manifestation as the cause of VCF was found in about 10%. [17]. Mukherjee et al. postulated an overall cancer diagnosis rate by transpedicular biopsies during kyphoplasty and vertebroplasty of 5.5% (6 of 109) when combining patients with no prior history of cancer or cancer thought to be in remission. Therefore, the authors recommended to perform a biopsy at each level in cases of multiple-level VCF, to avoid missing a malignancy [17].

There are also critical voices regarding biopsies in context with vertebroplasty and kyphoplasty. In a study by Hershkovich et al. only one case out of 99 biopsies (1.2%) with unsuspected malignancy could be detected [18]. Based on this observation, they calculated the cost for one diagnosed case of unsuspected malignancy around $31,000. Pneumaticos et al. recommend performing a bone biopsy only in patients where the preoperative evaluation raises the suspicion of a non-osteoporotic etiology as in their cohort the results of the biopsy only confirmed the diagnosis suspected from preoperative workup [19].

Beside these concerns, most studies recommend additional biopsy if a patient is treated by vertebroplasty or kyphoplasty to rule out a pathological fracture caused by malignancy with the aim to start specific therapy as early as possible.

Thirteen of 16 studies used preoperative MRI to assess the probability of a malignant cause for the VCF—sometimes in combination with CT and in three studies in combination with a bone scintigraphy (Tables 1 and 2). One study did not report on the modality of preoperative imaging. Studies combining a bone scan with an MRI had unexpected malignancies in 1–3% only [11, 18, 19], compared to 0.4–6% in studies using an MRI [8, 10, 12–17, 20, 21], and 6% in the one study reporting on unexpected lesions using CT only [22].

Studies published within the last three years (after 2018) reported unexpected malignancies in only 1% of the cases [13, 18, 20].

## Discussion

Transpedicular kyphoplasty and vertebroplasty are some of the most frequent spinal interventions and have become an established treatment option for vertebral compression fractures.

The differentiation between benign VCFs and pathologic fractures secondary to a malignant disease can be challenging. Magnetic resonance imaging (MRI), computed tomography (CT) and nuclear medical imaging like positron emission tomography (PET-CT) are well established for distinguishing between benign and malignant spinal diseases. In this context, Zhihong et al. demonstrated that malignant processes could be successfully diagnosed by preoperative MRI in almost 98% of patients with malignancy [20]. However, in many cases, a tissue diagnosis is needed for pathological confirmation.

The aim of this study was to summarize the existing knowledge on transpedicular biopsies during these interventions and their value in the detection of malignant diseases. It was the authors’ hypothesis that the prevalence of unexpected malignancies is higher than presumed by most surgeons.

This systematic review revealed sixteen articles reporting data from 3667 transpedicular biopsies in 3083 patients.

Within the analyzed studies, most biopsies confirmed osteoporosis as the dominant underlying pathology of VCFs. This systematic analysis of the current literature demonstrates that transpedicular biopsies reveal an unexpected malignancy in about 0.4–6% [8, 10–13, 15–20, 22].

Cancer represents a noticeable economic burden to our health care systems. An early diagnosis of a malignant disease can also reduce the costs of treatment and may reduce the economic impact on patients and their relatives due to shorter treatment and earlier return to work [23, 24]. Whether this is also true for unexpected cancer diagnoses after vertebral biopsies is unclear. Most spinal malignancies are metastatic and the cancer disease is by definition of advanced stage (stage IV). Hence, the benefit of an earlier diagnosis will be reduced compared to an earlier diagnosis in patients with stages I to III. A major benefit of an early diagnosis can be mainly be expected in rare primary tumors of the spine or in some patients with oligo-metastatic disease [25].

In addition, the value of cancer screening has been under debate because of the risk for false positives, which can result in unnecessary interventions, increased morbidity and mortality and a psychological burden to the patients [24].

In contrast to most cancer screening methods, a transpedicular biopsy has a much higher sensitivity and specificity. This means, in particular, that the likelihood for false positives and overdiagnosis is very low [24].

Furthermore, the estimated costs of about 200 Euros for the biopsy needle and for the histopathological assessment are relatively low and seem to be cost-effective in comparison to routine cancer screening campaigns [12].

A key limitation of this systematic review is the high percentage of retrospective studies included into the analysis, even though the results of the retrospective studies largely match the findings reported by the prospective studies. It was sometimes difficult to differentiate the definition of “unexpected” or “unsuspected” malignant lesions as it varied strongly with each study. This made a direct comparison between or an meta-analysis among all studies impossible. Hence, we cannot provide an averaged rate of unexpected malignancies for all 3667 transpedicular biopsies included but only a range from 0.4 to 6%.

While most studies used preoperative MRI to assess whether there was a malignant cause for the fracture, some studies combined MRI with a bone scan and other used CT only. Naturally, the type of preoperative imaging available will have influence on what we expect to be the outcome of a biopsy.

Most authors of the studies included into this systematic review recommend routine transpedicular biopsies during vertebroplasty or kyphoplasty based on the observed rates of unexpected malignant lesions. As stated before, however, even the definition of “unexpected” or “unsuspected” varies strongly among the analyzed studies. Hence, some authors suggest to do biopsies only in patients with a certain degree of suspicion [18, 19].

Combining the information from a patient’s history, the physical exam and laboratory tests and the information from different imaging modalities can increase the accuracy in preoperatively differentiating between malignancy and osteoporosis. The advances in medical imaging will increase our ability to correctly predict the presence of a malignant lesion as cause for a VCF [20, 26]. This will further decrease the role of routine transpedicular biopsies during kyphoplasty and vertebroplasty in future. This is underlined by the fact that studies using more modern imaging and studies published in the more recent past had noticeably lower rates of unexpected malignant findings in the biopsies.

However, in view of the considerably high incidence of unexpected malignant lesions, the authors recommend to routinely perform transpedicular biopsies during kyphoplasty or vertebroplasty of VCFs. With further advancement of modern tumor diagnostics and imaging, this recommendation will have to be re-evaluated in future, though.

Eventually, it remains a community-based decision to calculate the costs versus the benefits of routine transpedicular biopsies.

## Conclusion

Routine transpedicular biopsies during kyphoplasty or vertebroplasty reveal an unexpected malignant diagnosis in 0.4–6% of the cases, even though the definition of “unexpected” varies among the analyzed studies. The evidence to support a routine biopsy is inconsistent. Nevertheless, routine biopsies can be considered, especially when sufficient preoperatvie imaging is not available or radiological findings are unclear.
